# Supplementary figures and images for: The organization of serotonergic fibers in the Pacific angelshark brain: neuroanatomical and supercomputing analyses
Source: Front Neurosci. 2025 Aug 8;19:1602116. doi: 10.3389/fnins.2025.1602116 (PMC12370763; doi:10.3389/fnins.2025.1602116)

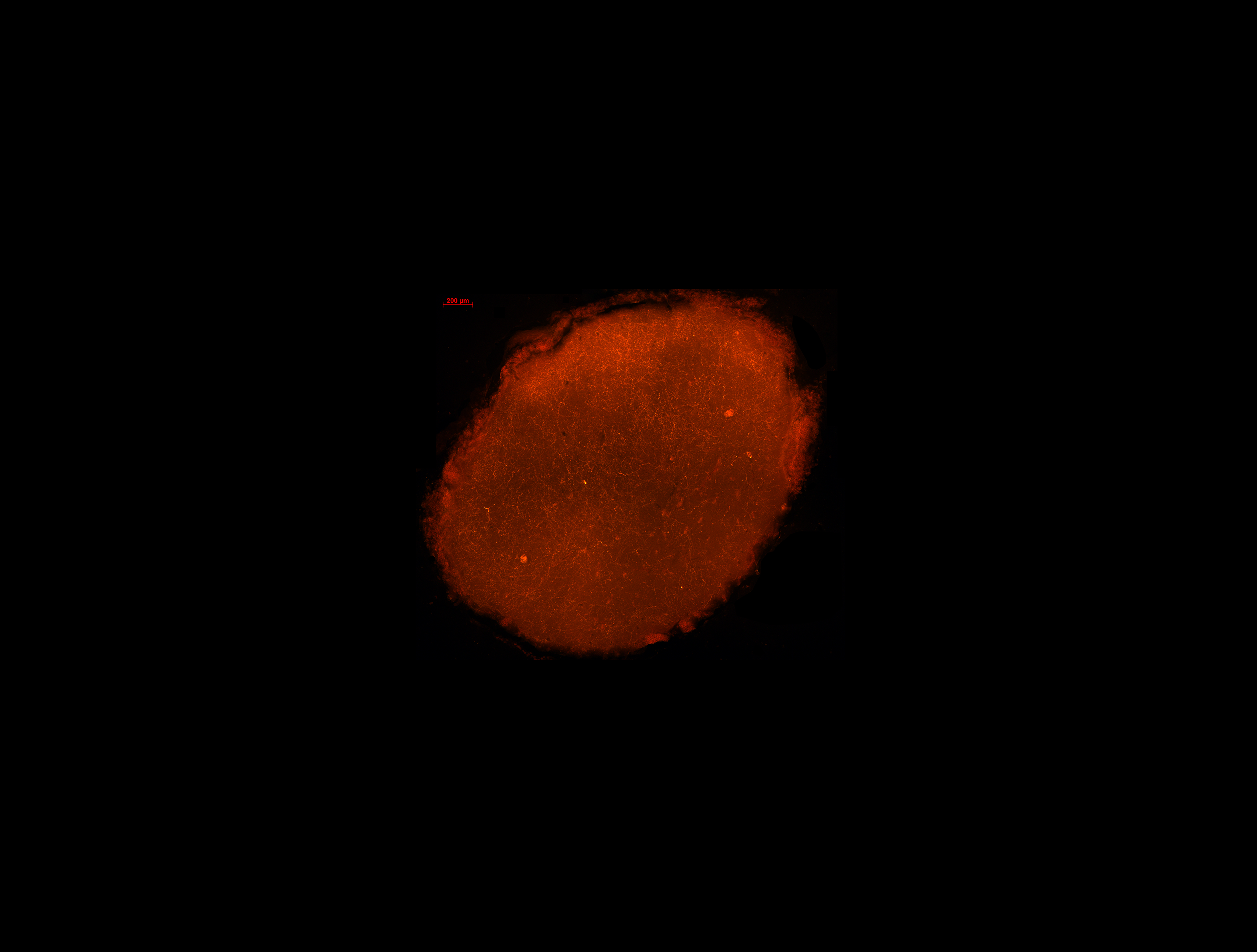

Supplement: Supplementary file 1 [file Image_1.tif]

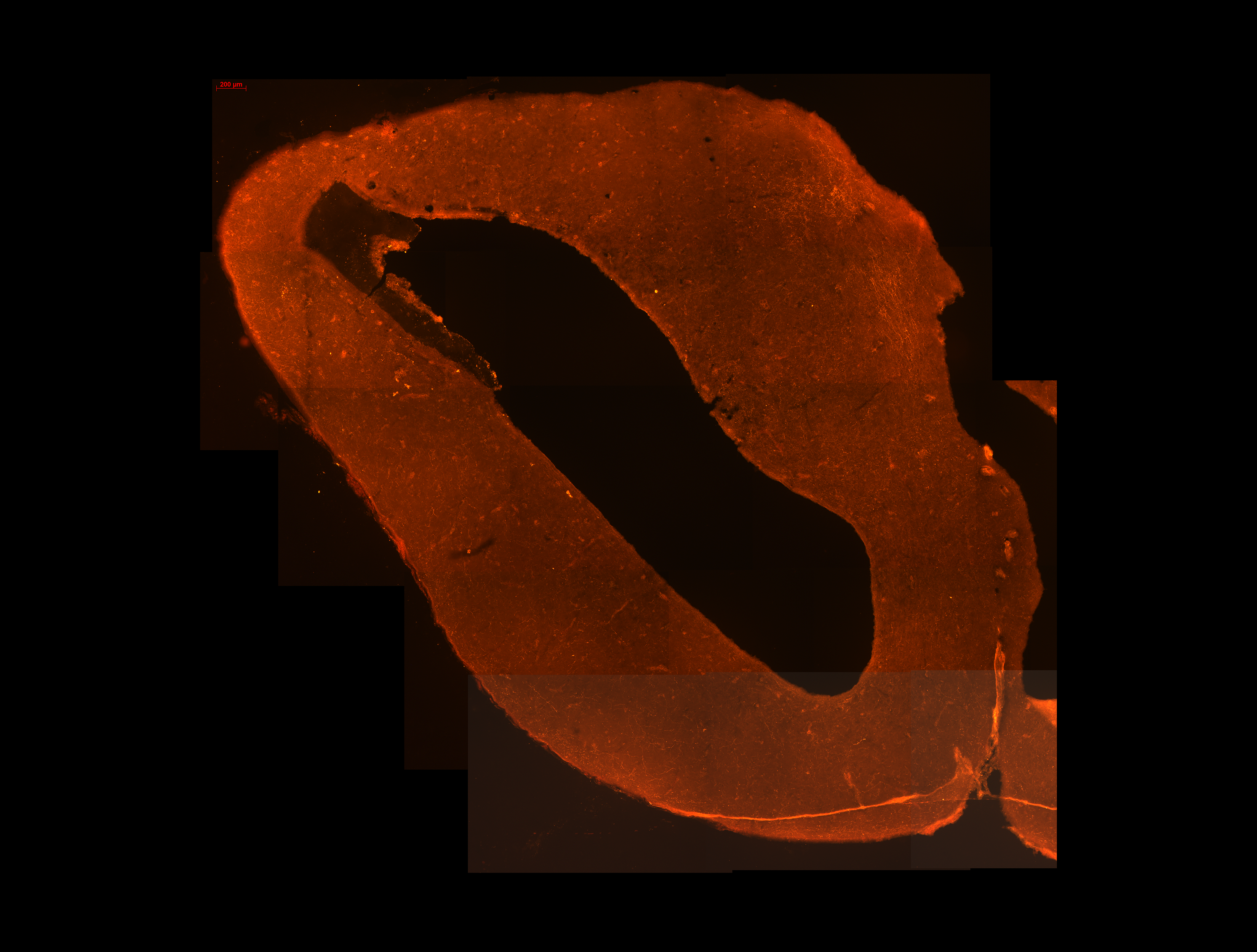

Supplement: Supplementary file 2 [file Image_2.tif]

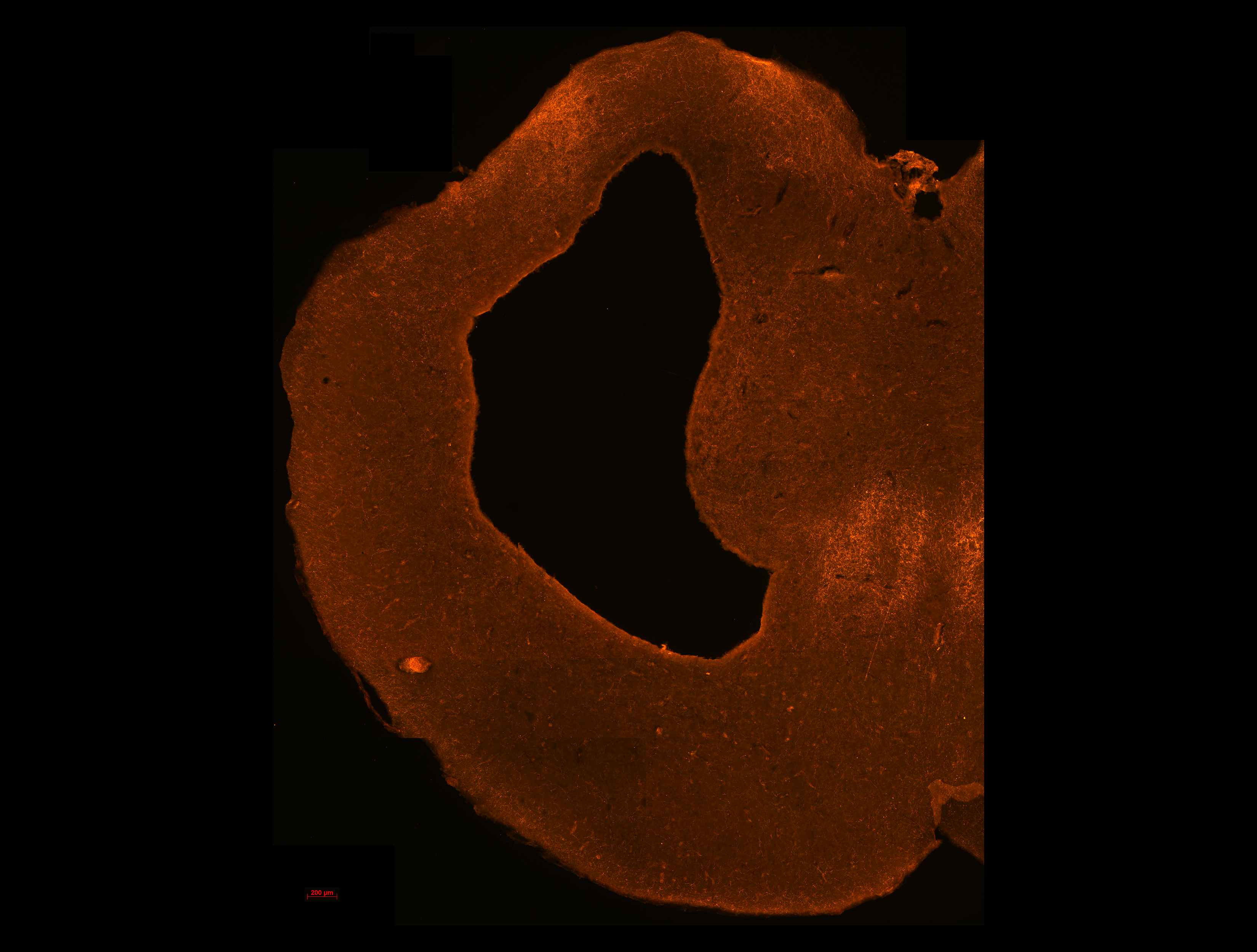

Supplement: Supplementary file 3 [file Image_3.tif]

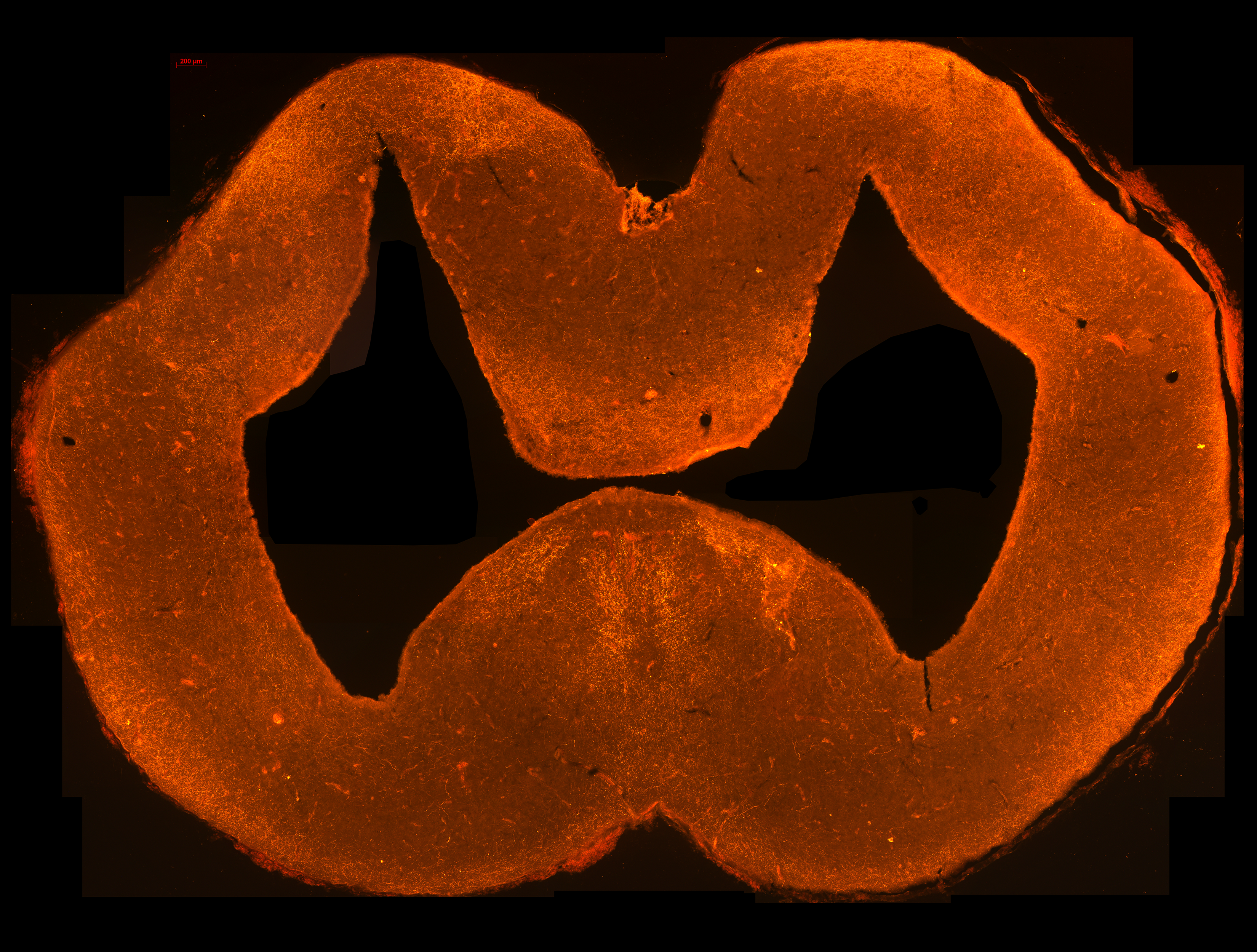

Supplement: Supplementary file 4 [file Image_4.tif]

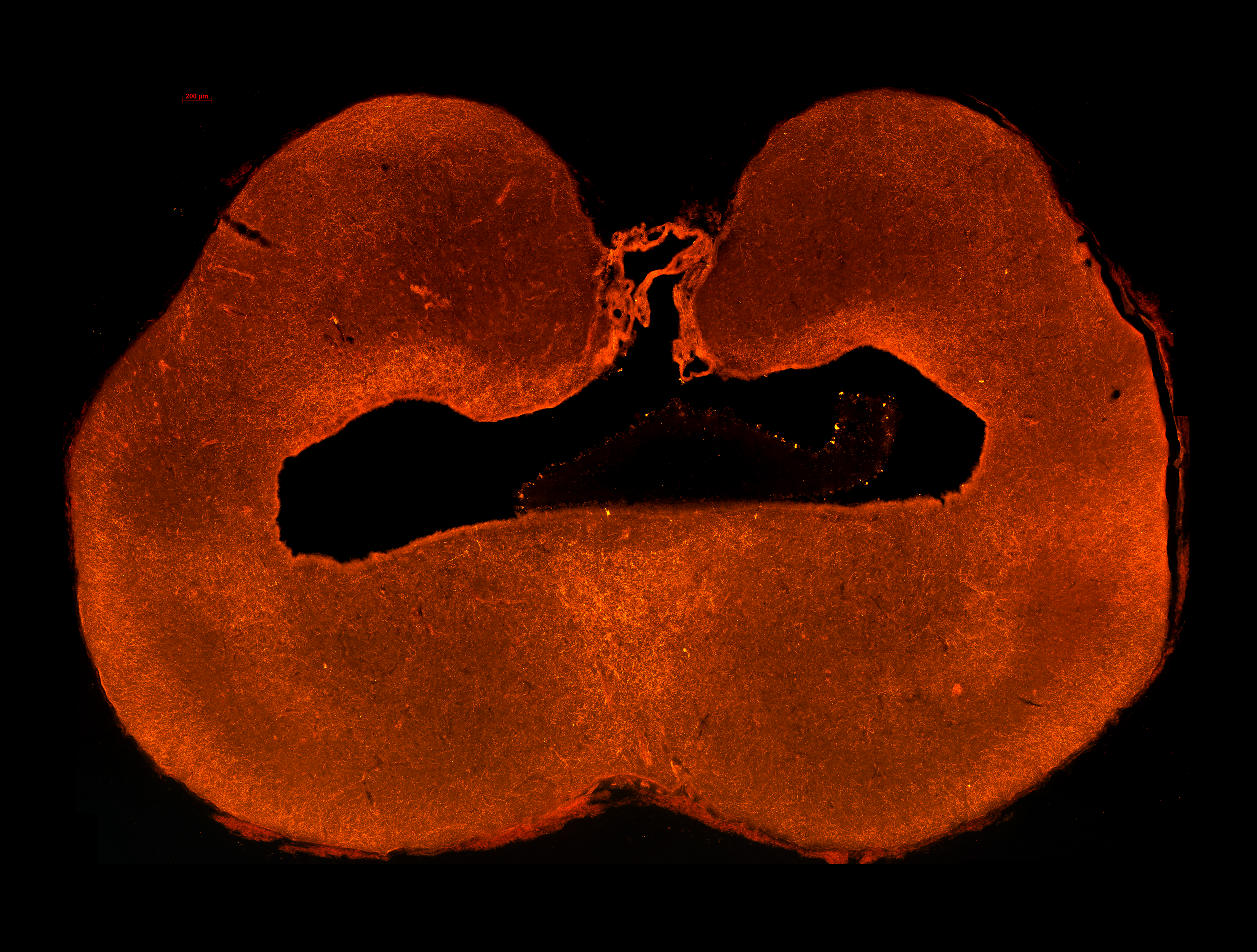

Supplement: Supplementary file 5 [file Image_5.tif]

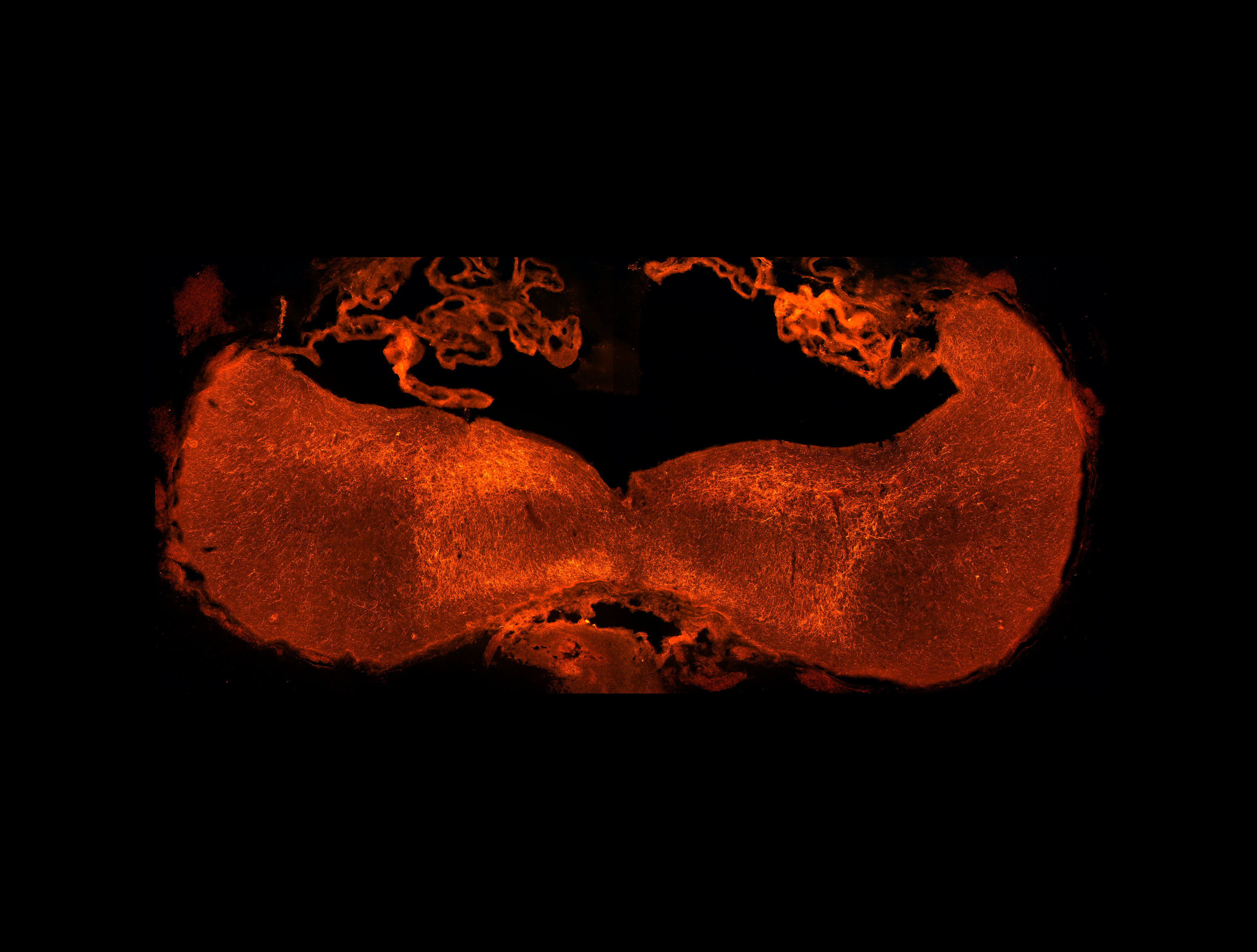

Supplement: Supplementary file 6 [file Image_6.tif]

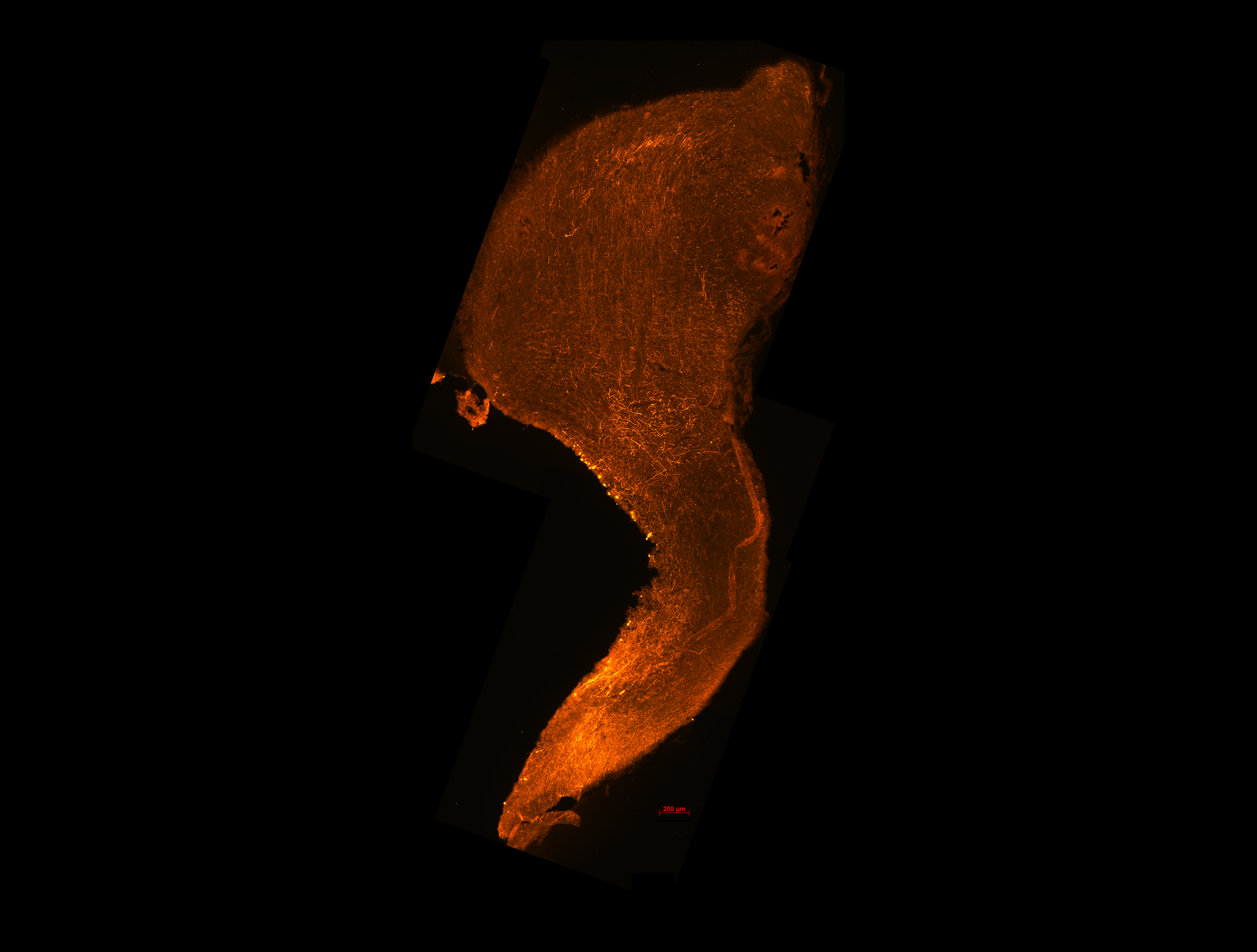

Supplement: Supplementary file 7 [file Image_7.tif]

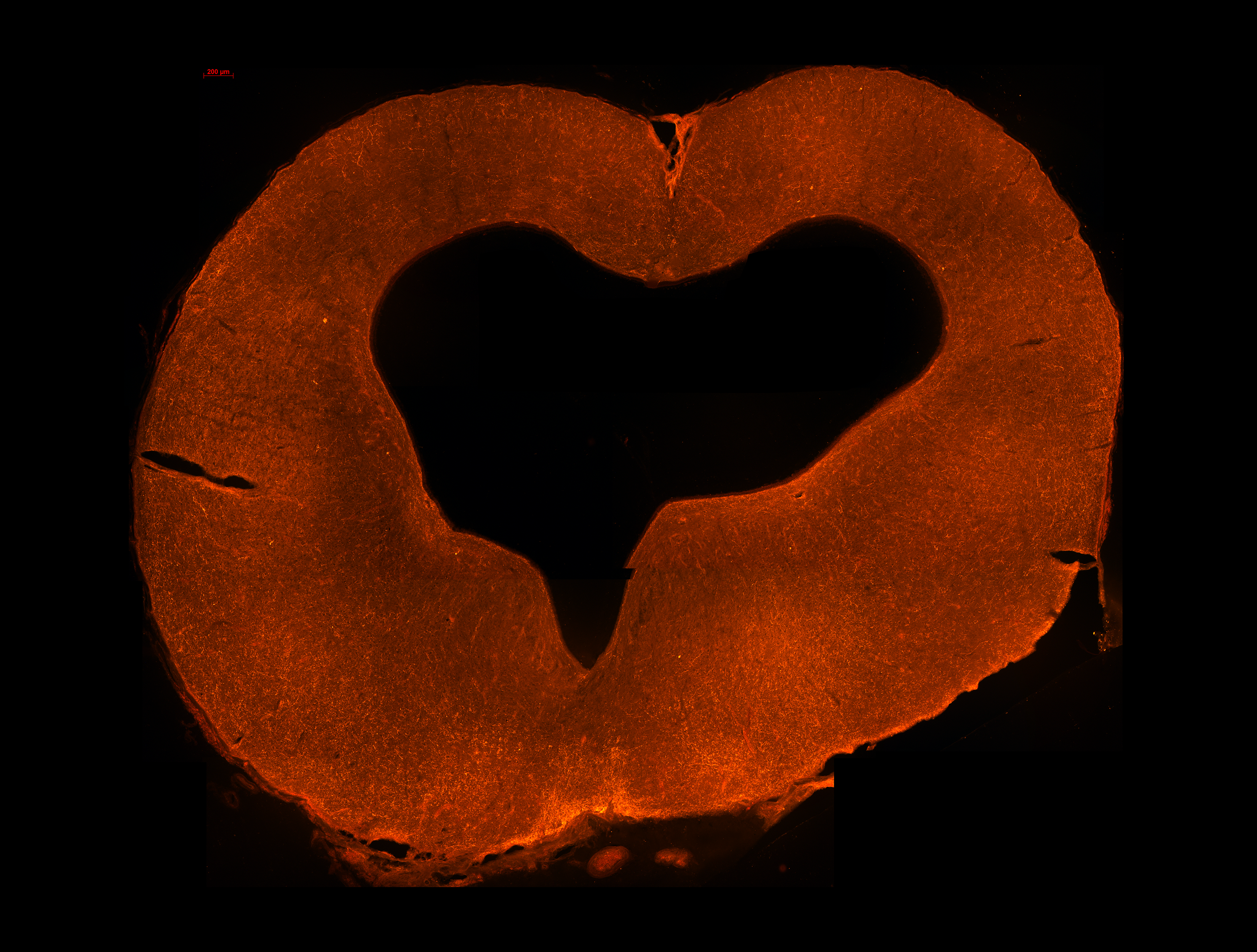

Supplement: Supplementary Figures 1–8 — High-resolution (tiled) images of serotonergic fibers at eight coronal levels of the Pacific angelshark telencephalon (corresponding to panels Figures 5A–H). The sections were immunostained for serotonin (5-HT) with the Cy3 fluorophore and imaged in color with a 5× objective. [file Image_8.tif]
